# Beneficial Influence of Water-Soluble PEG-Functionalized C_60_ Fullerene on Human Osteoblast Growth In Vitro

**DOI:** 10.3390/ma14061566

**Published:** 2021-03-22

**Authors:** Piotr Piotrowski, Katarzyna Klimek, Grazyna Ginalska, Andrzej Kaim

**Affiliations:** 1 Department of Chemistry, University of Warsaw, Pasteura 1 Street, 02-093 Warsaw, Poland; ppiotrowski@chem.uw.edu.pl (P.P.); akaim@chem.uw.edu.pl (A.K.); 2 Department of Biochemistry and Biotechnology, Medical University of Lublin, Chodzki 1 Street, 20-093 Lublin, Poland; g.ginalska@umlub.pl

**Keywords:** functionalized fullerenes, PEG, water solubility, cytotoxicity, cell proliferation, osteoblasts

## Abstract

The purpose of this study was to make an initial assessment of new PEG (polyethylene glycol)-functionalized C_60_ fullerene derivative for potential bone tissue engineering applications. Thus, Fourier Transform Infrared spectroscopy analysis, thermogravimetric analysis, and cyclic voltammetry measurement were performed. Moreover, cell culture experiments in vitro were carried out using normal human osteoblasts. Cell viability and proliferation were evaluated using colorimetric 3-(4,5-dimethylthiazol-2-yl)-2,5-diphenyltetrazolium bromide (MTT) test as well as by fluorescent staining. It was demonstrated that resultant derivative possessed good solubility in water, high temperature stability, and retained favorable electron accepting properties of C_60_ fullerene core. Most important, new fullerene derivatives at low concentrations did not exhibit cytotoxic effect and supported osteoblast proliferation compared to control. Thanks to all mentioned properties of new PEG-functionalized C_60_ fullerene derivative, it seems that it could be used as a component of polymer-based bone scaffolds in order to enhance their biological properties.

## 1. Introduction

Despite many years of research, fullerenes and their derivatives are still an interesting scientist subject due to their properties. Their use in medicine is particularly distinguished [1,2,3]. For this purpose, they usually need targeted functionalization as pristine fullerenes are nearly insoluble in physiological fluids. Hydroxylation of fullerenes, resulting in formation of so called fullerenols, are one of the most widely used method for synthesis of water-soluble derivatives for medicinal applications [4,5,6]. Nevertheless, due to their high degree of functionalization, in this case, fullerene core loses its characteristic beneficial properties. Another type of functionalized fullerenes that are extensively used in biomedical research are both C_60_ and C_70_ carboxyfullerenes. However, to obtain their high solubility, the introduction of multiple carboxylic acid groups is required [7,8,9]. The alternative approach, which allows to gain water solubility and functionalize fullerene cage by introduction of only one adduct, is modification with diverse poly(ethylene glycol) macromolecules [10].

To date, fullerenes have already found some interesting applications in medicine. For instance, it was found that carbonaceous materials, especially C_60_ fullerene and its derivatives, despite no solubility in physiological fluids, can be promising scaffolds for bone tissue engineering applications [11,12,13,14,15]. In the case of water-soluble derivates, fullerenes modified with poly(ethylene glycol) are especially used in photodynamic tumor therapy [16,17,18,19,20]. Moreover, recent research indicated that fullerenols nanoparticles can be considered as promising agents for osteoporosis treatment [21,22].

In this study, a water-soluble α-hydroxy-ω-C_60_ aziridinofullerene terminated poly(ethylene glycol) was synthesized and its physicochemical properties as well as its influence on osteoblast viability and proliferation in vitro were assessed. To our best knowledge, it is the first water-soluble C_60_ fullerene derivative designed for bone tissue engineering applications.

## 2. Materials and Methods

### 2.1. Materials

Bovine serum albumin (BSA), DMEM/Ham F12 medium, dimethyl sulfoxide solvent (DMSO), formaldehyde solution (36.5–38%), G418 disulfate salt solution, Hoechst 33342 fluorescent dye, hydrochloric acid (HCl), penicillin-streptomycin solution, phosphate buffered saline (PBS), sodium dodecyl sulfate (SDS), thiazolyl blue tetrazolium bromide (MTT), triton X-100, trypsin-ethylenediaminetetraacetic acid (trypsin-EDTA) solution (0.25%), KBr (FTIR grade), *o*-dichlorobenzene (ODCB), poly(ethylene glycol) *α*-hydroxy-*ω*-azido terminated (average M_n_ = 5000 Da), polyethylene glycol (PEG) (average M_n_ = 6000 Da) were supplied by Sigma-Aldrich Chemicals, Warsaw, Poland. Fullerene C_60_ powder (720.6 g/mol; 99.9% purity) was obtained from MER Corp., Tucson, AZ, USA. Fetal bovine serum (FBS) was purchased from Pan-Biotech, Aidenbach, Bavaria, Germany, while AlexaFluor^TM^ 635 Phalloidin from Invitrogen, Warsaw, Poland. Normal human fetal osteoblasts (hFOB 1.19 cell line, CRL-11372^TM^) were obtained from ATTC, Teddington, United Kingdom. 

### 2.2. Synthesis of C60NPEG_5000_

**Figure 1:**
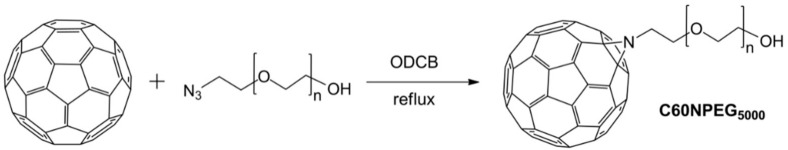
The scheme presenting synthesis of C60NPEG_5000_ fullerene derivative.

The *α*-hydroxy-*ω*-C_60_ aziridinofullerene terminated PEG_5000_ was synthesized using modified method reported by Yau et al. [10] (Figure 1). Ten-fold excess of fullerene was used to avoid formation of multi-adducts caused by high reactivity of azides and C_60_. Firstly, solution of *α*-hydroxy-*ω*-azido terminated poly(ethylene glycol) (125 mg, 0.025 mmol, average M_n_ = 5.000 Da) in 10 mL of ODCB was added dropwise to a well stirred mixture of C_60_ fullerene (180 mg, 0.25 mmol) in 15 mL of *o*-dichlorobenzene. Then, the mixture was refluxed and stirred for 24 h, after which the solvent was removed under reduced pressure. Water (100 mL) was added to resulting solid and obtained mixture was sonicated. Afterwards, the resultant solution was decanted from the precipitate, residue was centrifuged, and supernatant solution was added to soluble part. Then, it was concentrated on rotary evaporator to ca. 3–4 mL and precipitate of the product formed. After drying, C60NPEG_5000_ was obtained as brown solid 42 mg (Yield: 29%). ^1^H NMR (500 MHz, D_2_O) δ 4.80 (s, D_2_O), 4.03 (m, -OH), 4.72 (s, CH_2_) ppm; FT-IR (KBr disk) ʋ_max_(cm^−1^) 3395.9, 2966.6, 1670.4, 1498.7, 1432.1, 1386.9, 1255.0, 1094.4, 864.6, 829.1, 660.1, 576.6, 526.4.

### 2.3. Characterization of C60NPEG_5000_

Nuclear magnetic resonance (^1^H NMR) spectra were registered in D_2_O on Varian Unity Plus 500 MHz spectrometer. The Fourier Transform Infrared (FTIR) spectroscopy analysis was carried out in order to confirm successful functionalization of C60 fullerene with PEG. The samples were prepared as KBr disks and the measurements were performed using the Shimadzu 8400S spectrometer. The thermal stability of investigated samples was assessed by thermogravimetric analysis (TGA) using Q50 TGA (TA Instruments). The analyzed samples were previously dried under vacuum at 60 °C and the measurement was carried out under flow of nitrogen with heating rate of 10 K/min. In turn, electrochemical properties were evaluated using cyclic voltammetry. Cyclic voltammogram for PEG-functionalized fullerene was recorded at room temperature on Autolab PGSTAT 204 potentiostat, with three electrode arrangement: GC electrode (3 mm diameter) as working electrode, Ag/AgCl as the reference electrode and platinum wire as the counter electrode. The 0.1 M Bu_4_NPF_6_ in *o*-DCB:acetonitrile (3:1, *v*/*v*) was used as the supporting electrolyte solution.

### 2.4. Cell Culture Experiments

Prior to test, the solutions of C60NPEG_5000_ were prepared. Briefly, the samples of C60NPEG_5000_ (20 mg) were put into 1.5 mL tubes and subjected to sterilization by ethylene oxide. Then, they were dissolved in 200 µL of sterile distilled water (stock solution was 100 mg/mL). The C60NPEG_5000_ solutions were prepared by two-fold serial dilution method using cell culture medium—DMEM/Ham F12 medium supplemented with FBS at concentration of 2% (for cytotoxicity evaluation) or 10% (for cell proliferation assessment).

In order to evaluate cytotoxicity of C60NPEG_5000_, the osteoblasts (hFOB 1.19 cells) in complete culture medium were seeded on 96-well plates at concentration of 1.5 × 10^4^ cells/well. After 24-h incubation at 34 °C in a humidified incubator with 5% CO_2_, the culture medium was replaced by C60NPEG_5000_ solutions ranged from 1000–1.95 μg/mL. Culture medium + 2% FBS without C60NPEG_5000_ and 3% DMSO solution (prepared in culture medium + 2% FBS) were used as negative (NC) and positive (PC) controls of cytotoxicity, respectively. The culture medium with addition of 2% FBS was used to minimizing the cell divisions. The plates were transferred to the incubator for 48 h. In turn, to evaluate the influence of C60NPEG_5000_ on osteoblast proliferation, hFOB 1.19 cells in complete culture medium were seeded on 96-well plates at concentration of 3 × 10^3^ cells/well. After 24-h incubation at 34 °C in a humidified incubator with 5% CO_2_, the culture medium was replaced by selected dilution of PEG_5000_ functionalized C_60_ solutions (1.95; 7.81; 31.30; 125 μg/mL). As a control, culture medium + 10% FBS without C60NPEG_5000_ (0 μg/mL) was used. According to ATCC guidelines, culture medium supplemented with 10% FBS allows for proper cell proliferation. The plates were transferred to the incubator for 2- and 4-days.

Cell viability (after 48 h) and cell proliferation (after 2 and 4 days) were assessed using MTT assay according to procedure described previously [23]. The cell culture tests were performed in three independent experiments in octuplicate. The cell number during proliferation assay was determined using standard curve obtained for known number of cells. The cell doubling time was estimated based on data from proliferation assay and calculated using the following Equation (1):

(1) $$\mathrm{Cell}\;\mathrm{doubling}\;\mathrm{time}=\frac{(t_{2}-t_{1})\times \log\left(2\right)}{\left(\log Nt_{2}-\log Nt_{1}\right)}$$

where *t*_1_ denotes earlier time after cell seeding, *t*_2_ denotes later time after cell seeding, while *Nt*_1_ and *Nt*_2_ denote number of cells determined at these times.

The results were expressed as mean values ± SD. Resultant data were analyzed using unpaired *t*-test or one-way ANOVA test followed by Tukey’s multiple comparison test (GraphPad Prism 5, Version 5.04 Software).

Moreover, in order to confirm the results obtained with MTT test during proliferation assessment, cell morphology and density after 2- and 4-day incubation with the most promising concentration of C60NPEG_5000_ solution were visualized by confocal laser scanning microscopy (CLSM, Olympus Fluoview equipped with FV1000). The cell nuclei were stained with Hoechst 33342, while cell cytoskeleton with AlexaFluor^TM^ 635 Phalloidin.

## 3. Results and Discussion

### 3.1. Evaluation of Physicochemical Properties of C60NPEG_5000_

#### 3.1.1. H NMR and FTIR Measurements

**Figure 2:**
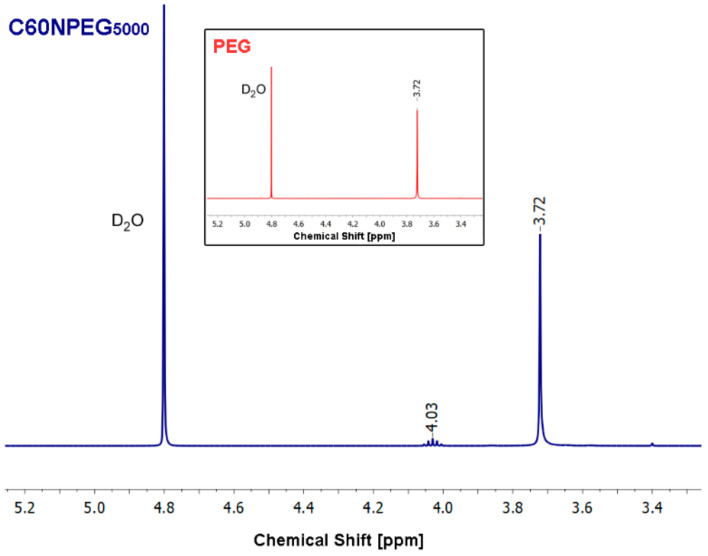
^1^H NMR spectrum of PEG-functionalized C_60_ fullerene and pure PEG (insert).

The NMR spectra recorded for analyzed samples dissolved in D_2_O are presented in Figure 2. Despite the signal coming from solvent, both signals expected for PEG-functionalized C_60_ fullerene were also observed. Firstly, multiplet attributed to the terminal hydroxyl group centered at 4.03 ppm was detected [24]. The next signal registered at 3.72 ppm confirmed presence of PEG chain in the structure of synthesized fullerene derivative [24,25]. Additionally, we have registered ^1^H NMR spectrum for pure PEG under the same conditions, which showed the same signal from –CH_2_ groups.

**Figure 3:**
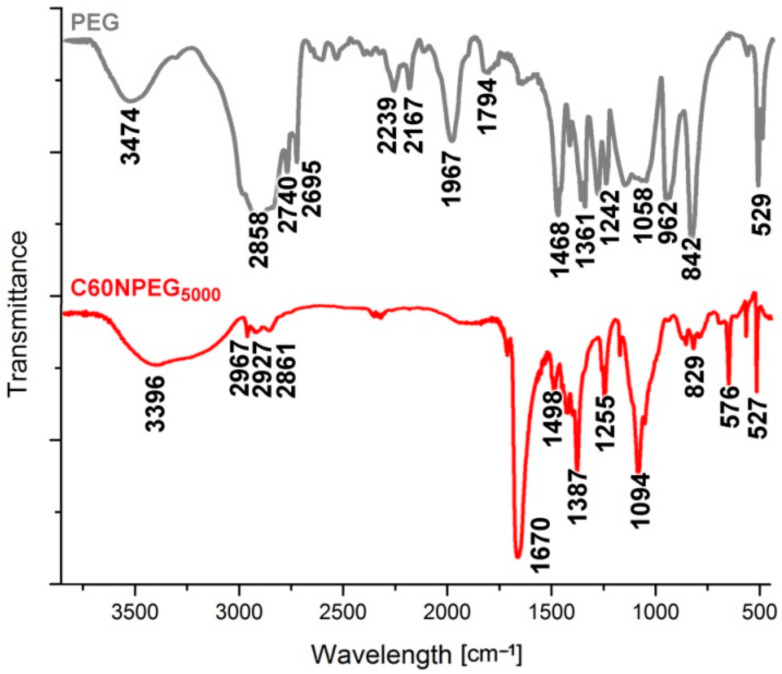
FTIR spectrum of C60NPEG_5000_ (red line) and unmodified PEG (grey line).

The registered FTIR spectrum showed broad signal centered at 3396 cm^−1^, which can be attributed to O-H stretching of terminal hydroxyl groups (Figure 3). The alkane C-H stretching signals were observed at 3967, 2927, and 2861 cm^−1^. Next signal, which was registered at 1670 cm^−1^ can be related to C=C stretching band or hydroxyl groups [26], and was reported previously in other PEG-functionalized fullerene derivatives [25]. Peak observed at 1387 cm^−1^ was associated with O-H bending mode. The C-O-C stretching band coming from PEG chain was registered at 1255 cm^−1^, along with typical ether C-O stretching vibration observed at 1094 cm^−1^. As expected, for C_60_ fullerene core sharp signals at 576 and 527 cm^−1^ were presented in the fingerprint region of the registered spectrum [27]. 

We have also registered FTIR spectrum for unmodified PEG, which shares numerous signals with the one obtained for fullerene derivative. The O-H stretching observed at 3474 cm^−1^, as well as presence of methylene C-H stretching at 2858, 2740, and 2695 cm^−1^. C-H bending mode was registered at 1468 cm^−1^, thus slightly shifted when compared to the same vibration in PEG-functionalized C_60_ (1498 cm^−1^). Signal observed at 1058 cm^−1^ can be attributed to the ether C-O-C bond in PEG chains.

#### 3.1.2. Thermogravimetric Analysis

**Figure 4:**
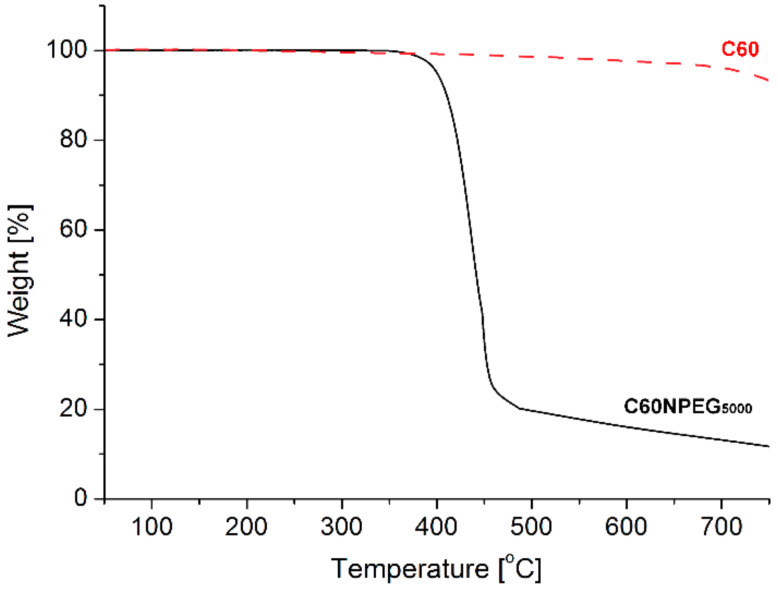
The TGA curves registered for C60NPEG_5000_ and C_60_ fullerene at 10K/min in N_2_ atmosphere.

The synthesized fullerene derivative revealed high thermal stability up to 350 °C, then it decomposed in the one major step ending around 480 °C (Figure 4), which is in good agreement with values reported previously [28,29]. Due to the fact that pristine fullerene remained stable over 600 °C under the same measurement conditions, we can conclude that observed weight loss is associated only with decomposition of PEG substituent. Total weight loss associated within this process was determined to be 81%, which is slightly lower than expected for mono-adducts of hydroxy terminated PEG_5000_. Thus, these results confirmed that the use of excess of C60 fullerene during the preparation of the C60NPEG_5000_ adduct allowed to prevent multiple addition reaction to its core. At higher temperatures slow decomposition of fullerene was observed. Lack of any other decomposition steps confirmed the total removal of the solvents, which was used during the synthesis and indicated high purity of the sample.

#### 3.1.3. Cyclic Voltammetry

**Figure 5:**
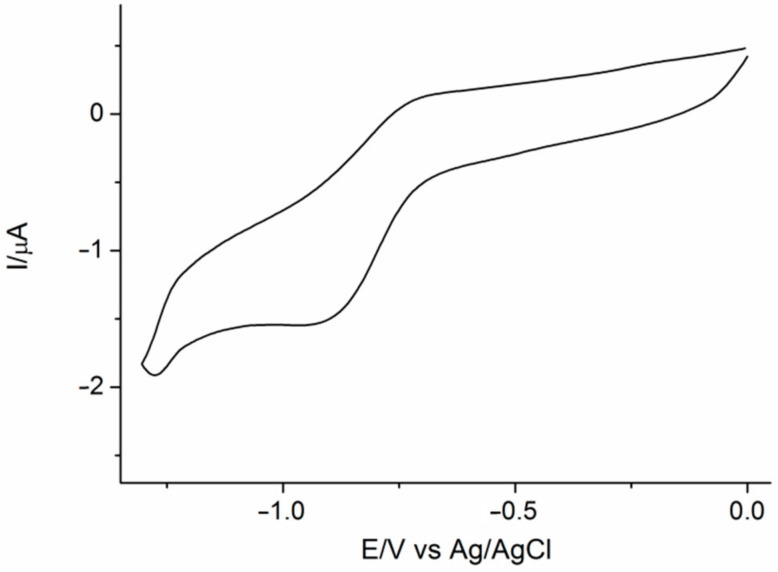
Cycylic voltammogram obtained for solution of C60NPEG_5000_ registered in 0.1 M Bu_4_NPF_6_ in o-DCB:acetonitrile (3:1, *v*/*v*) 4:1 (50 mV/s).

The registered voltammogram showed one quasi-reversible redox couple (Figure 5), corresponding to first step of reduction of C_60_ fullerene core. Redox potentials of this voltammetric peaks (E_pc_ = −0.89 V, E_pa_ = −0.72 V, E^0^ = −0.80 V) were shifted towards negative potentials, when compared to the reported for pristine C_60_ [30]. Thus, it confirmed chemical modification of fullerene core. Low intensity of observed signals can be attributed to moderate solubility of C60NPEG_5000_ in the supporting electrolyte solution, as well as a result of low fullerene to PEG ratio in synthesized molecule. The voltamperometric results indicated that the obtained adduct retained the reducing properties of fullerenes [31].

### 3.2. Evaluation of Biological Properties of C60NPEG_5000_

#### 3.2.1. Cytotoxicity towards Human Osteoblasts

**Figure 6:**
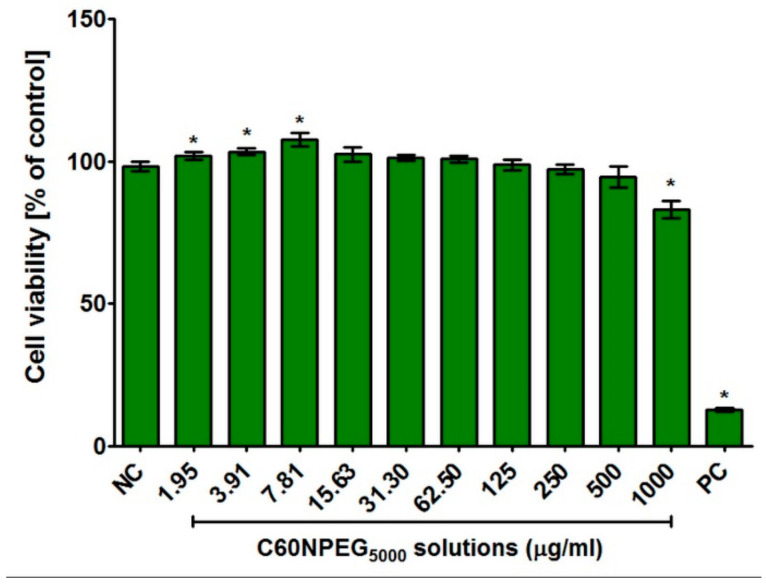
Human osteoblast viability (hFOB 1.19 cell line, ATCC CRL-11372^TM^) after 48-h incubation with C60NPEG_5000_ solutions. Culture medium without C60NPEG_5000_ was used as a negative control of cytotoxicity (NC), while 3% solution of DMSO was used as a positive control one (PC). The results were obtained using MTT assay. (* significantly different results compared to culture medium without C60NPEG_5000_-NC; unpaired *t*-test, *p* < 0.05).

The MTT assay demonstrated that most of tested concentrations of C_60_ fullerene derivative solutions were not cytotoxic towards human osteoblasts (Figure 6). Only significant decrease in cell viability to approx. 83% (*p* = 0.0014) was observed at the highest tested concentration (1000 μg/mL). Importantly, at low concentrations, namely 1.95–7.81 μg/mL, the cell viability was slightly but statistically higher (ranged from 102–108%, *p* < 0.05) compared to culture medium without C60NPEG_5000_ (negative control of cytotoxicity—NC). In turn, the viability of cells treated with 3% DMSO solution (positive control of cytotoxicity—PC) was approx. 12.70% (*p* < 0.0001) compared to the viability of cells incubated with culture medium without C60NPEG_5000_ (NC). It is worth mentioning that several research indicated that some water-soluble fullerene adducts had beneficial cytoprotective properties [32,33,34,35,36]. At low concentrations, they exhibited hepato- [34], cardio- [35], nephro- [33], and neuroprotective [36] activities thanks to their ability to scavenge free radicals [32]. Moreover, Zha et al. [36] demonstrated that water-soluble polyhydroxyfullerene (fullerenol) affected viability of hippocampal neurons in vitro in a concentration-dependent manner. It had positive effect on cell viability at low concentrations, followed by no negative effect and cytotoxic one at the highest tested concentrations. Thus, our results are in good agreement with data obtained by other authors, as we observed positive influence of low concentrations of water-soluble C60NPEG_5000_ derivative on viability of normal human osteoblasts.

#### 3.2.2. Proliferation of Human Osteoblasts

**Figure 7:**
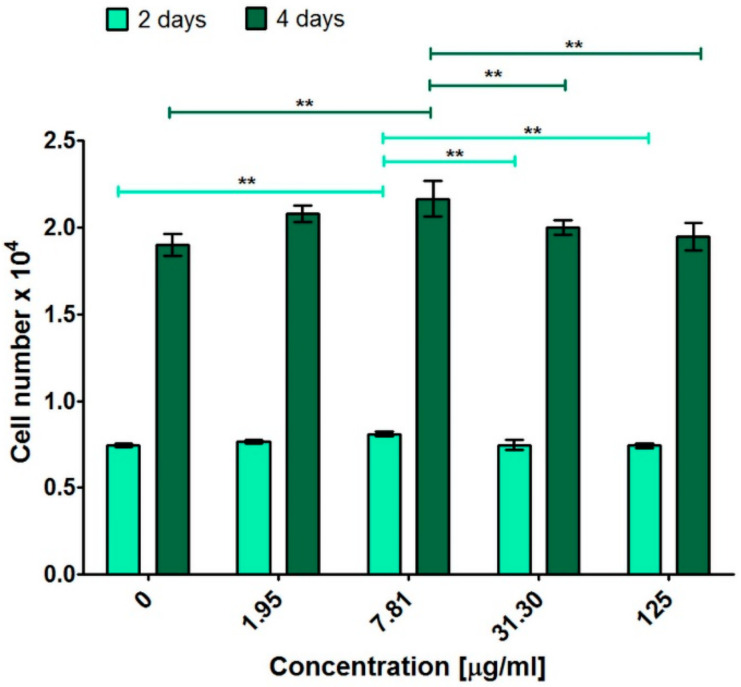
Human osteoblast proliferation (hFOB 1.19 cell line, ATCC CRL-11372^TM^) after 2- and 4-days incubation with C60NPEG_5000_ solutions. The results were obtained using MTT assay. Culture medium without C60NPEG_5000_ was used as a control of experiment (0 μg/mL). The data were analyzed using one-way ANOVA test followed by Tukey’s multiple comparison test (** *p* ≤ 0.01).

**Figure 8:**
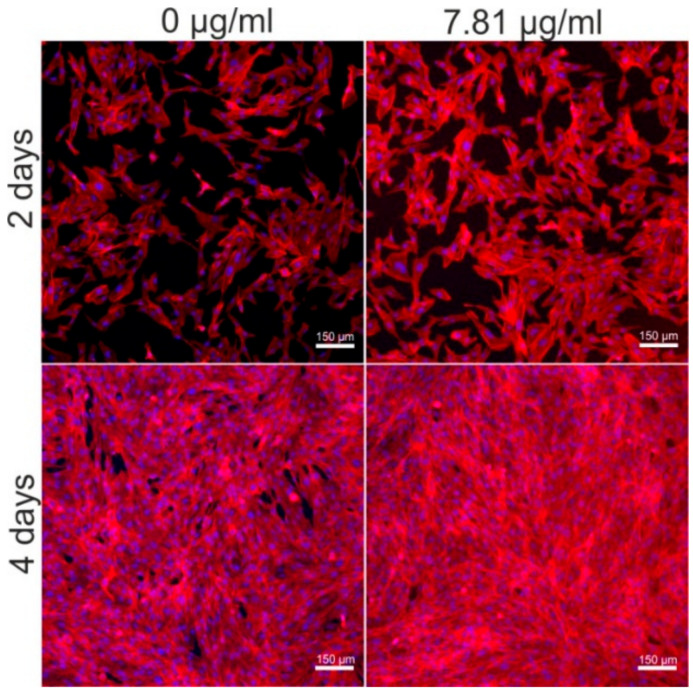
The confocal laser scanning microscope (CLSM) images showing osteoblast (hFOB 1.19 cell line, ATCC CRL-11372^TM^) nuclei (blue fluorescence) and F-actin filaments (red fluorescence) after 2- and 4-days incubation with C60NPEG_5000_ solution at concentration of 7.81 μg/mL. Culture medium without C60NPEG_5000_ (0 μg/mL) was used as a control. Magnification 100×, scale bar equals 150 μm.

In order to determine the influence of PEG_5000_ functionalized C_60_ fullerene on cell divisions, low-concentration solutions were selected and incubated with osteoblasts for 2- and 4-days. Performed colorimetric test (MTT assay) indicated that C60NPEG_5000_ solutions had ability to support osteoblast proliferation greater than control (culture medium without C60NPEG_5000_—0 μg/mL) (Figure 7 and Table 1). In particular, statistically significant results compared to control at both time points was obtained for C60NPEG_5000_ solution at concentration of 7.81 μg/mL (Figure 7). Confocal microscope observation confirmed these results and showed that after treatment with C60NPEG_5000_ solution at concentration of 7.81 μg/mL (Figure 8), the cells were in good condition (were flattened and well spread) as well as their density was higher compared to control cells. Thus, these data indicated that resultant fullerene derivative at low concentrations were not only non-toxic towards osteoblasts, but also promoted their growth and divisions. The studies performed by other research groups demonstrated the beneficial influence of non-water-soluble C_60_ layers on adhesion, proliferation, and differentiation of osteoblast-like cells (MG-63 cell line) [12,13,15]. In turn, Geng et al. revealed that water-soluble fullerenol nanoparticles had ability to enhance proliferation of bone marrow macrophages (BMM) [21]. Data obtained in our study revealed positive effect of water-soluble fullerene derivative (C60NPEG_5000_) on human osteoblast response in vitro. Interestingly, it was found that physicochemical and biological properties of fullerenes depend on the applied procedure for their functionalization [3]. Presumably, the beneficial biological effect of C60NPEG_5000_ derivative resulted from its functionalization by PEG terminated with hydroxyl group. Similar effect was observed for graphene oxide (GO) functionalized with amino-terminated PEG chains (PEG-GO). It was demonstrated that modification of GO using PEG with polar terminal group significantly improved its biodistribution and stability, which led to increase of cell growth, proliferation, and differentiation [37]. Nevertheless, to determine precise mechanism of action of C60NPEG_5000_ on osteoblast growth and proliferation, additional research is needed.

## 4. Conclusions

New PEG-functionalized C_60_ fullerene derivative was synthesized using 1,3-dipolar cycloaddition of corresponding azide, followed by subsequent elimination of nitrogen. Resultant product revealed good solubility in water, high temperature stability, and retained favorable electron accepting properties of C_60_ fullerene core. Importantly, some solutions of new fullerene derivative were not only non-toxic towards normal human osteoblasts (hFOB 1.19 cells), but they also had the ability to promote the viability of these cells. Moreover, at low concentrations, C_60_ fullerene derivative enhanced osteoblast proliferation, which indicates its promising potential as biomaterial for bone tissue engineering applications.

## Figures and Tables

**Table 1:** Average values of doubling time determined for human osteoblasts (hFOB 1.19 cell line, ATCC CRL-11372^TM^) after incubation with selected concentrations of C60NPEG_5000_ solution. Cells incubated with culture medium without C60NPEG_5000_ (0 μg/mL) was used as a control of experiment.

Sample	Average Cell Doubling Time (Days)
Culture medium without C60NPEG_5000_ (0 μg/mL)	1.481
C60NPEG_5000_ 1.95 μg/mL	1.405
C60NPEG_5000_ 7.81 μg/mL	1.390
C60NPEG_5000_ 31.30 μg/mL	1.407
C60NPEG_5000_ 125 μg/mL	1.436

## Data Availability

Data available on reasonable request.

## References

[1] Bakry R., Vallant R.M., Najam-ul-Haq M., Rainer M., Szabo Z., Huck C.W., Bonn G.K. (2007). Medicinal applications of fullerenes. Int. J. Nanomed..

[2] Castro E., Hernandez Garcia A., Zavala G., Echegoyen L. (2017). Fullerenes in Biology and Medicine. J. Mater. Chem. B.

[3] Goodarzi S., Da Ros T., Conde J., Sefat F., Mozafari M. (2017). Fullerene: Biomedical engineers get to revisit an old friend. Mater. Today.

[4] Grebowski J., Kazmierska P., Krokosz A. (2013). Fullerenols as a new therapeutic approach in nanomedicine. Biomed. Res. Int..

[5] Rašović I. (2017). Water-soluble fullerenes for medical applications. Mater. Sci. Technol..

[6] Nakamura E., Isobe H. (2003). Functionalized Fullerenes in Water. The First 10 Years of Their Chemistry, Biology, and Nanoscience. Acc. Chem. Res..

[7] Ali S.S. (2012). Carboxyfullerenes: Nanomolecules that Work!. J. Nanomed. Biother. Discov..

[8] Liu Q., Zhang X., Zhang X., Zhang G., Zheng J., Guan M. (2013). C70-Carboxyfullerenes as Efficient Antioxidants to Protect Cells against Oxidative-Induced Stress. ASC Appl. Mater. Interfaces.

[9] Dugan L.L., Turetsky D.M., Du C., Lobner D., Wheeler M., Almli C.R., Shen C.K.-F., Luh T.-Y., Choi D.W., Lin T.-S. (1997). Carboxyfullerenes as neuroprotective agents. Proc. Natl. Acad. Sci. USA.

[10] Yau H.C., Bayazit M.K., Steinke J.H.G., Shaffer M.S.P. (2014). Diamond rings or dumbbells: Controlling the structure of poly(ethylene glycol)-fullerene [60] adducts by varying linking chain length. Macromolecules.

[11] Eivazzadeh-Keihan R., Maleki A., de la Guardia M., Bani M.S., Chenab K.K., Pashazadeh-Panahi P., Baradaran B., Mokhtarzadeh A., Hamblin M.R. (2019). Carbon based nanomaterials for tissue engineering of bone: Building new bone on small black scaffolds: A review. J. Adv. Res..

[12] Vandrovcova M., Vacik J., Svorcik V., Slepicka P., Kasalkova N., Vorlicek V., Lavrentiev V., Vosecek V., Grausova L., Lisa V. (2008). Fullerene C 60 and hybrid C 60/Ti films as substrates for adhesion and growth of bone cells. Phys. Status Solidi Appl. Mater. Sci..

[13] Kopova I., Lavrentiev V., Vacik J., Bacakova L. (2015). Growth and potential damage of human bone-derived cells cultured on fresh and aged C60/Ti films. PLoS ONE.

[14] Vandrovcová M., Bačáková L. (2011). Adhesion, growth and differentiation of osteoblasts on surface-modified materials developed for bone implants. Physiol. Res..

[15] Grausova L., Vacik J., Bilkova P., Vorlicek V., Svorcik V., Soukup D., Bacakova M., Lisa V., Bacakova L. (2008). Regionally-selective adhesion and growth of human osteoblast-like MG 63 cells on micropatterned fullerene C60 layers. J. Optoelectron. Adv. Mater..

[16] Tabata Y., Murakami Y., Ikaya Y. (1997). Photodynamic effect of polyethylene glycol-modified fullerene on tumor. Jpn. J. Cancer Res..

[17] Liu J., Ohta S.I., Sonoda A., Yamada M., Yamamoto M., Nitta N., Murata K., Tabata Y. (2007). Preparation of PEG-conjugated fullerene containing Gd3+ ions for photodynamic therapy. J. Control. Release.

[18] Asada R., Liao F., Saitoh Y., Miwa N. (2014). Photodynamic anti-cancer effects of fullerene [C60]-PEG complex on fibrosarcomas preferentially over normal fibroblasts in terms of fullerene uptake and cytotoxicity. Mol. Cell. Biochem..

[19] Liao F., Saitoh Y., Miwa N. (2011). Anticancer effects of fullerene [C60] included in polyethylene glycol combined with visible light irradiation through ROS generation and DNA fragmentation on fibrosarcoma cells with scarce cytotoxicity to normal fibroblasts. Oncol. Res..

[20] Tabata Y., Murakami Y., Ikada Y. (1997). Antitumor Effect of Poly(Ethylene Glycol)-Modified Fullerene. Fuller. Sci. Technol..

[21] Geng H., Chang Y.N., Bai X., Liu S., Yuan Q., Gu W., Li J., Chen K., Xing G., Xing G. (2017). Fullerenol nanoparticles suppress RANKL-induced osteoclastogenesis by inhibiting differentiation and maturation. Nanoscale.

[22] Chen K., Geng H., Liang W., Liang H., Wang Y., Kong J., Zhang J., Liang Y., Chen Z., Li J. (2020). Modulated podosome patterning in osteoclasts by fullerenol nanoparticles disturbs the bone resorption for osteoporosis treatment. Nanoscale.

[23] Klimek K., Belcarz A., Pazik R., Sobierajska P., Han T., Wiglusz R.J., Ginalska G. (2016). “False” cytotoxicity of ions-adsorbing hydroxyapatite—Corrected method of cytotoxicity evaluation for ceramics of high specific surface area. Mater. Sci. Eng. C.

[24] Mahou R., Wandrey C. (2012). Versatile route to synthesize heterobifunctional poly(ethylene glycol) of variable functionality for subsequent pegylation. Polymer.

[25] Collavini S., Saliba M., Tress W.R., Holzhey P.J., Völker S.F., Domanski K., Turren-Cruz S.H., Ummadisingu A., Zakeeruddin S.M., Hagfeldt A. (2018). Poly(ethylene glycol)–[60]Fullerene-Based Materials for Perovskite Solar Cells with Improved Moisture Resistance and Reduced Hysteresis. ChemSusChem.

[26] Liu J., Chen R.Q., Wang C.P., Zhao Y.J., Chu F.X. (2019). Synthesis and characterization of polyethylene glycol-phenol-formaldehyde based polyurethane composite. Sci. Rep..

[27] Ruoff R.S., Kadish K.M., Boulas P., Chen E.C.M. (1995). Relationship between the electron affinities and half-wave reduction potentials of fullerenes, aromatic hydrocarbons, and metal complexes. J. Phys. Chem..

[28] Vrandečić N.S., Erceg M., Jakić M., Klarić I. (2010). Kinetic analysis of thermal degradation of poly(ethylene glycol) and poly(ethylene oxide)s of different molecular weight. Thermochim. Acta.

[29] Chieng B.W., Ibrahim N.A., Yunus W.M.Z.W., Hussein M.Z. (2014). Poly(lactic acid)/poly(ethylene glycol) polymer nanocomposites: Effects of graphene nanoplatelets. Polymer.

[30] Echegoyen L., Echegoyen L.E. (1998). Electrochemistry of Fullerenes and Their Derivatives. Acc. Chem. Res..

[31] Tam J., Liu J., Yao Z. (2013). Effect of microstructure on the antioxidant properties of fullerene polymer solutions. RSC Adv..

[32] Sharoyko V.V., Ageev S.V., Podolsky N.E., Petrov A.V., Litasova E.V., Vlasov T.D., Vasina L.V., Murin I.V., Piotrovskiy L.B., Semenov K.N. (2021). Biologically active water-soluble fullerene adducts: Das Glasperlenspiel (by H. Hesse)?. J. Mol. Liq..

[33] Injac R., Boskovic M., Perse M., Koprivec-Furlan E., Cerar A., Djordjevic A., Strukelj B. (2008). Acute doxorubicin nephrotoxicity in rats with malignant neoplams can be successfully treated With fullerenol C60(OH)24 via suppresion of oxidative stress. Pharmacol. Rep..

[34] Injac R., Perse M., Obermajer N., Djordjevic-Milic V., Prijatelj M., Djordjevic A., Cerar A., Strukelj B. (2008). Potential hepatoprotective effects of fullerenol C60(OH)24 in doxorubicin-induced hepatotoxicity in rats with mammary carcinomas. Biomaterials.

[35] Injac R., Perse M., Boskovic M., Djordjevic-Milic V., Djordjevic A., Hvala A., Cerar A., Strukelj B. (2008). Cardioprotective effects of fullerenol C60(Oh)24 on a single dose doxorubicin-induced cardiotoxicity in rats with malignant neoplasm. Technol. Cancer Res. Treat..

[36] Zha Y.Y., Yang B., Tang M.L., Chen J.T., Wen L.P., Wang M. (2012). Concentration-dependent effects of fullerenol on cultured hippocampal neuron viability. Int. J. Nanomed..

[37] Ghosh S., Chatterjee K. (2020). Poly(Ethylene glycol) functionalized graphene oxide in tissue engineering: A review on recent advances. Int. J. Nanomed..

